# Supplementary material for: Impact of Marker Pruning Strategies Based on Different Measurements of Marker Distance on Genomic Prediction in Dairy Cattle
Source: Animals (Basel). 2021 Jul 2;11(7):1992. doi: 10.3390/ani11071992 (PMC8300388; doi:10.3390/ani11071992)
Supplement: Supplementary file 1 [file animals-11-01992-s001.zip › animals-1224898-supplementary.pdf]

animals

MDPI

Article

Impact of Marker Pruning Strategies Based on Different Measurements of Marker Distance on Genomic Prediction in Dairy Cattle

Duanyang Ren, Jinyan Teng, Shuqi Diao, Qing Lin, Jiaqi Li and Zhe Zhang *

Supplementary Material

Table S1. Number of single nucleotide polymorphism (SNP) in each chromosome for high-density SNP data after quality control.

Chromosome number	Number of SNP	Chromosome number	Number of SNP
1	22,375	16	10,871
2	17,723	17	10,645
3	16,768	18	9,803
4	16,399	19	9,161
5	14,817	20	10,381
6	17,712	21	9,461
7	15,351	22	9,039
8	12,427	23	6,992
9	13,933	24	8,870
10	14,003	25	6,387
11	15,217	26	7,231
12	11,299	27	6,238
13	8,874	28	6,408
14	9,762	29	6,459
15	11,147		

Table S2. The ratios or thresholds used in the physical distance (PhyD), genetic distance (GenD) and random distance (RanD) methods.

SNP number levels	The ratio of selected markers (%) in the RanD method	The threshold of physical distance (bp) in the PhyD method	The threshold of r^2 in the GenD method
1k	0.3	2500000	0.00032
2k	0.6	1200000	0.00112
3k	0.9	800000	0.0025
4k	1.2	600000	0.0045
5k	1.5	480000	0.007
7k	2.1	333333	0.0155
10k	3	225000	0.045
15k	4.5	140000	0.18
20k	6	105000	0.43
30k	9	65000	0.78
50k	15	35000	0.975
75k	22.5	19900	0.9999
100k	30	12000	-
200k	60	3300	-

Table S3. Four scenarios of marker sets with the same mean and different variances for the physical distance between adjacent SNPs.

	mean	variance
Scenario 1	498816.6	63967021601
Scenario 2	498539.2	86119095978
Scenario 3	498123.8	142436000000
Scenario4	498403.6	271461357847

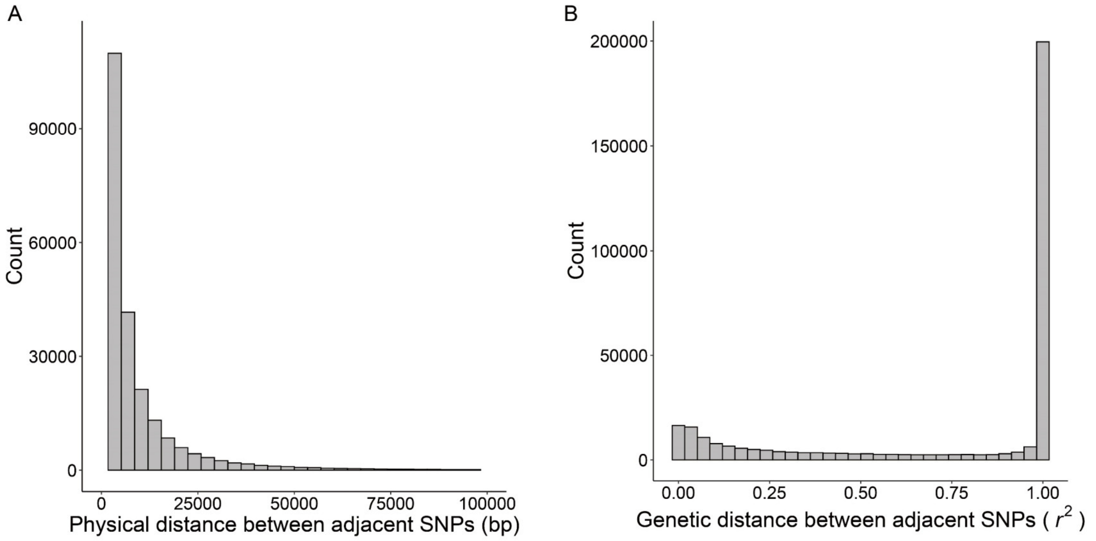

Figure S1. The distribution of the physical distance (A) and genetic distance (B) between adjacent SNPs. The physical and genetic distances are expressed in base pairs (bp) and r^2, respectively.

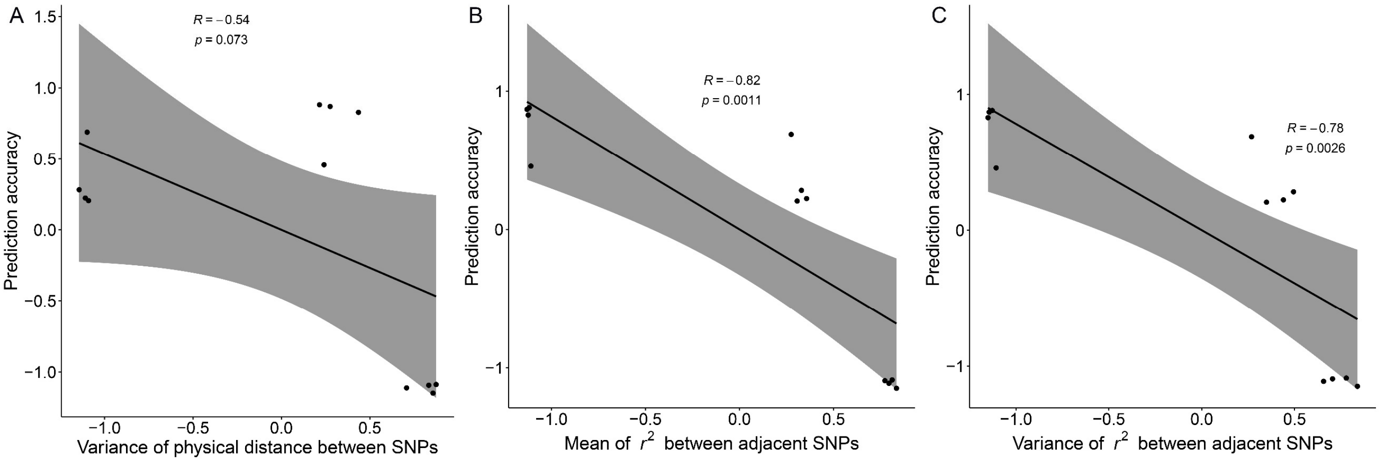

Figure S2. The correlation tests between normalized marker density-related measurements and the normalized genomic prediction accuracy in SCS. Correlation tests were performed by combining normalized marker density-related measurements and normalized GEBV accuracies on 15-50 k SNPs panels. The correlation coefficient, *P*-value of the correlation coefficient generated by each correlation test were given in the plot. The correlation test results of variance of the physical distance between adjacent SNPs, the mean and variance of the r^2 (genetic distance) between adjacent SNPs with GEBV accuracy are listed in (**A–C**) respectively.
